# Assessment of the nasal microbiota in dogs with fungal rhinitis before and after cure and in dogs with chronic idiopathic rhinitis

**DOI:** 10.1186/s12866-023-02828-7

**Published:** 2023-04-15

**Authors:** Emilie Vangrinsven, Aline Fastrès, Bernard Taminiau, Frédéric Billen, Georges Daube, Cécile Clercx

**Affiliations:** 1 Department of Clinical Sciences, FARAH, Faculty of Veterinary Medicine, University of Liège, Liège, Belgium; 2 Department of Food Sciences - Microbiology, FARAH, Faculty of Veterinary Medicine, University of Liège, Liège, Belgium

**Keywords:** Nasal cavity, Dog, Nasal, Microbiota, Aspergillosis, Fungal rhinitis, Mycotic rhinitis, Chronic idiopathic rhinitis

## Abstract

**Background:**

Pathogenesis of canine fungal rhinitis is still not fully understood. Treatment remains challenging, after cure turbinate destruction may be associated with persistent clinical signs and recurrence of fungal rhinitis can occur. Alterations of the nasal microbiota have been demonstrated in dogs with chronic idiopathic rhinitis and nasal neoplasia, although whether they play a role in the pathogenesis or are a consequence of the disease is still unknown. The objectives of the present study were (1) to describe nasal microbiota alterations associated with fungal rhinitis in dogs, compared with chronic idiopathic rhinitis and controls, (2) to characterize the nasal microbiota modifications associated with successful treatment of fungal rhinitis. Forty dogs diagnosed with fungal rhinitis, 14 dogs with chronic idiopathic rhinitis and 29 healthy control dogs were included. Nine of the fungal rhinitis dogs were resampled after successful treatment with enilconazole infusion.

**Results:**

Only disease status contributed significantly to the variability of the microbiota. The relative abundance of the genus *Moraxella* was decreased in the fungal rhinitis (5.4 ± 18%) and chronic idiopathic rhinitis (4.6 ± 8.7%) groups compared to controls (51.8 ± 39.7%). Fungal rhinitis and chronic idiopathic rhinitis groups also showed an increased richness and α-diversity at species level compared with controls. Increase in unique families were associated with fungal rhinitis (Staphyloccaceae, Porphyromonadaceae, Enterobacteriaceae and Neisseriaceae) and chronic idiopathic rhinitis (Pasteurellaceae and Lactobacillaceae). In dogs with fungal rhinitis at cure, only 1 dog recovered a high relative abundance of Moraxellaceae.

**Conclusions:**

Results confirm major alterations of the nasal microbiota in dogs affected with fungal rhinitis and chronic idiopathic rhinitis, consisting mainly in a decrease of *Moraxella*. Besides, a specific dysbiotic profile further differentiated fungal rhinitis from chronic idiopathic rhinitis. In dogs with fungal rhinitis, whether the NM returns to its pre-infection state or progresses toward chronic idiopathic rhinitis or fungal rhinitis recurrence warrants further investigation.

## Background

With advances in culture-independent technologies, the role of the upper respiratory tract microbiota in health and disease has become an intense area of research in human medicine [1–6] and to a much lesser extend in canine medicine [7–12].

Fungal rhinitis secondary to infection with *Aspergillus fumigatus* is a common cause of nasal disease in dogs while it is uncommon in humans. Why young otherwise healthy dolichocephalic dogs have their nasal cavity and sinus invaded by the fungus is not yet totally understood, despite extensive investigation [13–15]. The host itself but also the fungus have been described as contributing to local dysimmunity. The host develops a Th1 type mucosal immune response along with the up-regulation of proinflammatory cytokines and chemokines [16, 17]. In return the fungus reacts with in-host adaptive genetic and phenotypic changes which could enable growth of the pathogen and contribute to the suppression of the local immune system [15, 18].

In human beings, reports over the past five years suggest that the microbiota can modulate the host immune response to invading fungal micro-organisms [19–21]. The microbiota is expected to influence immune homeostasis through host-to-microbe and microbe-to-microbe interactions [4, 22–24]. It has been shown, by correlating changes in metabolite profiles with microbiota metagenomic composition, that certain bacterial species contribute to host-fungal symbiosis and mucosal homeostasis in humans with *Aspergillus*-related lung disease [25]. Formation of sinonasal bacterial biofilms of multiple species (such as *Staphylococcus aureus*, *Staphylococcus epidermidis* and *Pseudomonas aeruginosa*) have been reported to damage epithelia sufficiently for the establishment of fungal biofilms [26] while the release of extracellular molecules by *P. aeruginosa* has been shown to stimulate the growth of *A. fumigatus *[27]. It can therefore be suspected that, in the nasal cavities of dogs with fungal rhinitis, the microbiota is able to influence the immunological response to fungi, the clinical fungal disease severity, as well as the response to treatment.

Chronic idiopathic rhinitis is a common heterogeneous disease characterized by lymphoplasmacytic to mixed inflammation of the sinonasal cavities without any identifiable cause [28]. In humans, disruption of the microbiota homeostasis has been described as being the primary driver or at least exacerbating factor for nasal chronic inflammatory diseases such as chronic idiopathic rhinitis [2, 6, 24, 29–32] and allergic rhinitis [2, 6]. In the pathophysiology of chronic idiopathic rhinitis in humans, two other interesting notions are the importance of bacterial biofilms [33] as well as the concept of keystone species maintaining a stable and healthy state by providing resistance to colonization by pathogens [31].

Altogether, this suggests that specific bacterial strains, as well as factors influencing the microbial composition and/or modulating microbial disturbances may be an untapped source of therapeutics to mitigate the severity of upper respiratory tract infections and/or inflammation. However, the role of nasal microbiota (NM) alterations in the pathophysiology of canine chronic nasal diseases has been very little studied since only one single study describes alterations of the NM in dogs with nasal neoplasia or chronic rhinitis [10].

So far, data relative to the NM in dogs with fungal nasal disease are not available. Therefore, whether bacterial dysbiosis exist in fungal rhinitis, and whether it may be one of the factors able to either initiate or entertain the local fungal development, or allow recurrence, is unknown. Likewise, all therapeutic protocols for fungal rhinitis described are based on attempts to eliminate the fungus [13, 34, 35] instead of targeting the relationship between the host and the fungus, and a possible approach based on NM modulation has not been considered yet.

For all these reasons, further knowledge concerning the alterations of the NM in canine chronic nasal diseases such as fungal rhinitis and chronic idiopathic rhinitis is needed. Improved understanding of the relationships between the microbiota, host responses and non-native microorganisms would help to develop future therapeutic approaches acting to prevent the invasion of pathogenic microorganisms. A first step is to characterize any specific dysbiosis associated with both chronic idiopathic rhinitis and fungal rhinitis. Therefore, the aim of this study was to describe and compare the NM in dolichocephalic dogs with fungal rhinitis (at diagnosis and cure in a subpopulation of dogs) and in dogs with chronic idiopathic rhinitis versus a control population of healthy dogs.

## Results

### Study population

Eighty-three client-owned dogs were recruited and divided into 3 groups: 29 in the control group, 40 dogs in the fungal rhinitis (FR) group and 14 in the chronic idiopathic rhinitis (CR) group (Table 1). A first batch (7 healthy, 9 FR and 8 CR dogs) was collected and sequenced in 2017 [36], a second batch (22 healthy dogs and 17 dogs with FR) in 2018 and finally a last group was analyzed in 2020 (6 dogs with CR and 14 dogs with FR among which 9 dogs were sampled twice: at diagnosis and at cure). The data of the three sequencing sets were gathered in one table and processed together in one unique table. Before this step the same swabs, DNA extraction sets and primers sets were used and samples were manipulated in the same lab by the same technician.

**Table 1:** Characteristics of the groups according to the disease status

	**Control group**	**CR group**	**FR group**
Number	29	14	40
Age (years)	7 (0.8–11.3) ^a^	9 (1–14.3) ^a^	6.4 (1.2–14.3) ^a^
Gender	12 males, 17 females	6 males, 8 females	27 males, 14 females
Weight (kg)	30 (14.8–47.8) ^a^	24 (6–36) ^a^	30.2 (3.7–55) ^a^
Antibiotic treatment	/	1 (topical)	11 (systemic)
Antifungal treatment	/	/	10

*FR*: fungal rhinitis, *CR*: chronic idiopathic rhinitis

^a^Median (min–max)

In the control group, breeds included were Labrador retriever (*n* = 4), Belgian shepherd (*n* = 4), Border collie (*n *= 4), Australian shepherd (*n* = 4), Beauceron (*n* = 3), Golden retriever (*n* = 3), Alaskan malamute (*n* = 2), German shepherd (*n* = 1), Doberman (*n* = 1), Dalmatian (*n* = 1), White Swiss shepherd (*n* = 1), and mixed-breed (*n* = 1). Included dogs had a normal general examination and bloodwork and were not receiving any treatment within one month before sampling.

Breeds included in the FR group were Border collie (*n* = 5), rottweiler (*n* = 3), American Staffordshire terrier (*n* = 3), mixed-breed (*n* = 3), Labrador (*n* = 2), Bull Terrier (*n* = 1), Greater Swiss Mountain dog (*n* = 1), Golden retriever (*n* = 11), Cocker spaniel (*n* = 1), Great Dane (*n* = 1), Australian shepherd (*n* = 1), giant poodle (*n* = 1), Rhodesian ridgeback (*n* = 1), Beauceron (*n* = 1), Siberian husky (*n* = 1), German shepherd (*n* = 1), Dobermann (*n* = 1), Jack Russel (*n* = 1) and Dachshund (*n* = 1). At the time of sampling, 11 dogs were receiving systemic antimicrobials (Table 1), 10 dogs were treated with oral antifungal drugs, 4 with non-steroidal anti-inflammatory drugs and 2 with steroids, within the 2 previous weeks.

Breeds included in the CR group were mixed-breed (*n* = 3), Siberian husky (*n* = 2), Jack Russel terrier (*n* = 2), Dalmatian (*n* = 1), American Staffordshire terrier (*n* = 1), poodle (*n* = 1), Bernese mountain dog (*n* = 1), Barzoï (*n* = 1), Dachshund (*n* = 1) and Border collie (*n* = 1). One dog was treated with topical antimicrobial therapy (thiamphenicol) and saline at the time of sampling (Table 1). All the other dogs did not receive anti-inflammatory or antimicrobial treatment for at least two weeks before sampling albeit this was not an exclusion criterion.

### Nasal microbiota analysis

At the finest taxonomic level 4,887 operational taxonomic units (OTUs) were present throughout all samples. The Good’s coverage of all samples was higher than 96% with median 99.3% (96.6%-99.9%) indicating that the sequencing depth was sufficient for reliable analysis of these nasal microbial community samples. The distribution of age, sex and bodyweight according to disease status is reported in Table 1. Age did not differ significantly between groups (*p* = 0.15).

#### Healthy dogs

The most common taxa at phylum level were Proteobacteria (mean relative percentage 54.1%, min 1.0%-max 99.9%), Firmicutes (15.5%, 0.1–96.8%), Tenericutes (8.7%, 0.0–81.5%) and Actinobacteria (7.8%, 0.0–83.7%), representing 97% of the bacterial population in this group (Table 2). Beside the family Moraxellaceae, three dogs had a high relative abundance (> 50%) of Cardiobacteriaceae (phylum Proteobacteria) and two dogs a high relative abundance of an unclassified family of the Mollicutes class (phylum Tenericutes). Among the phylum Proteobacteria, the genus *Moraxella* represented the most abundant taxon with a mean relative percentage at 51.8%.

**Table 2:** Bacterial groups at > 1% mean relative abundance among the control, FR and CR groups at phylum, family and genus level

Taxon	**Control group (*n* = 29)**				**CR group (*n* = 14)**				**FR group (*n* = 40)**			
**Phylum** **Family** *Genus*	Mean rel. freq. (%)	SD (%)	Tukey's multiple comparisons test (corrected *p* < 0.05)	Detected in n dogs	Mean rel. freq. (%)	SD (%)	Tukey's multiple comparisons test (corrected *p* < 0.05)	Detected in n dogs	Mean rel. freq. (%)	SD (%)	Tukey's multiple comparisons test (corrected *p* < 0.05)	Detected in n dogs
**Proteobacteria**	71.6%	29.3	**A**	29	30.3%	22.2	B***	14	34.2%	29.8	B***	42
**Moraxellaceae**	52.0%	39.5	**A**	29	5.4%	9.6	B***	13	6.3%	18.0	B***	36
*Moraxella*	51.8%	39.7	**A**	29	4.6%	8.7	B***	11	5.4%	18.0	B***	32
**Neisseriaceae**	0.4%	1.4	**A**	12	7.1%	9.9	**AB**	13	7.7%	11.6	B*	34
*Conchiformibius*	0.3%	1.4	**A**	8	4.4%	8.7	**AB**	12	6.7%	11.3	B*	28
*Neisseria*	0.0%	0.1	**A**	3	2.6%	6.2	B*	9	1.0%	2.3	**AB**	25
**Enterobacteriaceae**	0.9%	2.8	NS	15	3.6%	6.1	NS	13	9.2%	21.9	NS	36
*Escherichia_Shigella*	0.3%	1.1	NS	11	1.7%	2.8	NS	13	7.0%	18.1	NS	35
*Proteus*	0.0%	0.0	NS	1	1.4%	5.4	NS	2	0.8%	5.1	NS	7
**Pasteurellaceae**	0.1%	0.4	A***	11	10.5%	14.5	**B**	13	1.6%	2.5	A***	29
*Pasteurella*	0.0%	0.0	A**	1	6.0%	13.4	**B**	11	0.1%	0.3	A**	19
*Pasteurellaceae_ge*	0.1%	0.4	A**	6	3.9%	7.5	**B**	9	1.2%	2.3	A*	26
**Cardiobacteriaceae**	9.8%	23.9	A*	19	0.1%	0.2	**AB**	6	0.4%	1.6	**B**	15
*Suttonella*	9.8%	23.9	A*	19	0.0%	0.0	**AB**	2	0.4%	1.6	**B**	7
**Pseudomonadaceae**	6.1%	12.5	NS	26	0.6%	1.1	NS	13	6.2%	20.4	NS	31
*Pseudomonas*	6.1%	12.5	NS	25	0.6%	1.1	NS	13	6%	20.4	NS	30
**Firmicutes**	10.3%	17.3	**A**	29	39.1%	27.2	B**	14	35.1%	31.9	B**	41
**Peptostreptococcaceae**	0.0%	0.1	NS	5	0.6%	0.9	NS	10	1.0%	2.5	NS	31
**Lactobacillaceae**	1.6%	5.3	**A**	10	19.6%	23.9	B*	14	9.4%	18.4	**AB**	28
*Lactobacillus*	1.6%	5.3	**A**	10	19.6%	23.9	B*	14	9.4%	18.4	**AB**	28
**Lachnospiraceae**	0.1%	0.2	**A**	8	0.8%	1.3	**AB**	11	1.1%	1.7	B*	32
**Bacillales_Family_XI**	0.1%	0.7	A***	15	1.3%	1.6	**B**	11	0.3%	0.5	A***	28
*Gemella*	0.1%	0.7	A***	1	1.3%	1.6	**B**	9	0.3%	0.5	A***	14
**Streptococcaceae**	4.0%	12.4	**A**	22	7.7%	6.0	B**	14	5.1%	6.3	**AB**	35
*Lactococcus*	0.4%	1.3	**A**	6	5.3%	6.3	B**	14	2.5%	4.4	**AB**	27
*Streptococcus*	3.6%	12.7	NS	20	2.4%	2.7	NS	12	2.6%	4.3	NS	34
**Staphylococcaceae**	2.1%	4.4	NS	20	4.4%	7.6	NS	11	14.1%	29.4	NS	39
*Staphylococcus*	2.1%	4.4	NS	19	4.4%	7.6	NS	11	13.9%	29.3	NS	39
**Bacteroidetes**	2.0%	5.6	**A**	26	13.4%	11.7	B*	14	13.4%	14.8	B***	41
**Porphyromonadaceae**	0.7%	3.5	**A**	5	4.0%	7.3	**AB**	10	6.5%	12.5	B*	35
*Porphyromonas*	0.7%	3.5	**A**	5	4.0%	7.3	**AB**	10	6.5%	12.5	B*	35
**Bacteroidaceae**	0.0%	0.1	NS	4	0.4%	0.5	NS	8	1.3%	3.9	NS	30
*Bacteroides*	0.0%	0.1	NS	5	0.4%	0.5	NS	8	1.3%	3.9	NS	30
**Flavobacteriaceae**	0.1%	0.4	**A**	10	4.7%	11.3	B*	10	1.1%	1.8	**AB**	29
*Capnocytophaga*	0.0%	0.2	NS	3	3.2%	7.5	NS	9	0.2%	0.4	NS	22
*Flavobacterium*	0.0%	0.2	NS	9	1.4%	4.6	NS	8	0.8%	1.5	NS	22
**Prevotellaceae**	0.0%	0.1	NS	8	0.5%	0.8	NS	9	1.0%	2.7	NS	28
**Weeksellaceae**	1%	3.0	NS	6	3.3%	5.7	NS	8	2.2%	4.0	NS	19
*Elizabethkingia*	0.0%	0.0	NS	1	2.3%	4.7	NS	6	1.1%	2.6	NS	16
**Actinobacteria**	5.4%	9.7	NS	29	12.5%	23.8	NS	14	8.5%	9.9	NS	36
**Corynebacteriaceae**	0.5%	1.1	NS	21	9.1%	24.2	NS	12	1.5%	3.2	NS	25
*Corynebacterium_1*	0.2%	0.4	NS	13	2.2%	5.0	NS	8	1.1%	3.0	NS	22
**Microbacteriaceae**	2.3%	4.6	NS	21	0.6%	0.9	NS	10	0.9%	1.4	NS	27
*Leucobacter*	2.0%	4.4	NS	19	0.4%	0.9	NS	8	0.4%	1.1	NS	15
**Micrococcaceae**	1.6%	7.5	NS	19	0.7%	1.5	NS	11	1.1%	2.4	NS	26
*Micrococcus*	1.4%	7.3	NS	8	0.3%	1.0	NS	6	0.1%	0.4	NS	14
**Propionibacteriaceae**	0.3%	0.8	NS	14	0.6%	0.8	NS	12	1.9%	3.5	NS	27
*Cutibacterium*	0.3%	0.8	NS	12	0.5%	0.6	NS	9	1.7%	3.4	NS	25
**Actinomycetaceae**	0.1%	0.5	**A**	5	0.8%	1.5	**AB**	10	1.0%	1.8	B*	26
*Actinomyces*	0.1%	0.5	NS	5	0.8%	1.5	NS	10	1.0%	1.8	NS	26
**Tenericutes**	9.7%	21.3	NS	18	2%	5.4	NS	10	1.9%	6.4	NS	29
**Mollicutes_fa**	9.5%	20.9	**A**	15	1.8%	4.8	**AB**	8	1.0%	6.2	B*	5
*Mollicutes_ge*	9.5%	20.9	**A**	15	1.8%	4.8	**AB**	8	1.0%	6.2	B*	3
**Fusobacteria**	0.1%	0.3	NS	6	1.6%	2.5	NS	11	4.2%	12.5	NS	34
**Fusobacteriaceae**	0.0%	0.2	NS	6	1.2%	1.9	NS	11	4.0%	12.5	NS	32
*Fusobacterium*	0.0%	0.2	NS	6	1.2%	1.9	NS	11	4.0%	12.5	NS	32

Mean relative percentages (mean rel. freq) and standard deviation (SD) of the most abundant bacterial groups, annotated to the level of phylum, family and genus, based on sequencing of the V1-V3 region of the 16S rRNA gene

A, B: operational taxonomic units not sharing a common letter differ significantly

*FR* fungal rhinitis, *CR* chronic idiopathic rhinitis

^*^p < 0.05;

^**^p < 0.01;

^***^p < 0.001; NS not significant

#### Dogs with fungal rhinitis at diagnosis and cure

At diagnosis, the most common taxa at phylum level were Firmicutes (mean relative percentage 35.1%, min 0.0%-max 99.8%) followed by Proteobacteria (34.2%, 0.1–98.6%) and Bacteroidetes (13.4, 0.0–60.4).

**Fig. 1:**
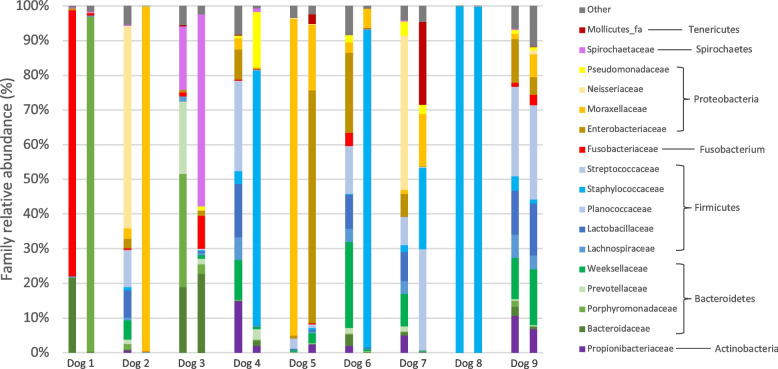
Composition of nasal microbiota at family level in dogs with fungal rhinitis at diagnosis (first bar) and cure (second bar) (other: mean relative frequency < 1%)

Out of the 40 dogs with FR, 9 were resampled at the time of cure. Median time to achieve cure in this subpopulation was 4.4 weeks (2.9–14). Six, 2 (dogs 5 and 6) and 1 (dog 2) dogs achieved cure after 1, 2 and 3 infusion protocols (Fig. 1).

The most common taxa at phylum level at the time of cure was equally distributed compared to diagnosis with Firmicutes (42.4%, 0.4–99.7%), Proteobacteria (27.0%, 0.1–99.3%) and Bacteroidetes (16.1%, 0.1–96.8%).

#### Dogs with chronic idiopathic rhinitis

At phylum level the most common taxa were Firmicutes (mean relative percentage 39.1%, min 0.1%-max 92.3%), Proteobacteria (30.3%, 0.7–75.4%), Bacteroidetes (13.4%, 0.3–40.7%) and Actinobacteria (12.5%, 0.0–97.8%).

### Comparison between healthy dogs and dogs with chronic nasal diseases

The bacterial load quantified by 16S rRNA gene quantitative polymerase-chain reaction (qPCR) did not differ between the three groups.

#### Constrained ordination

**Fig. 2:**
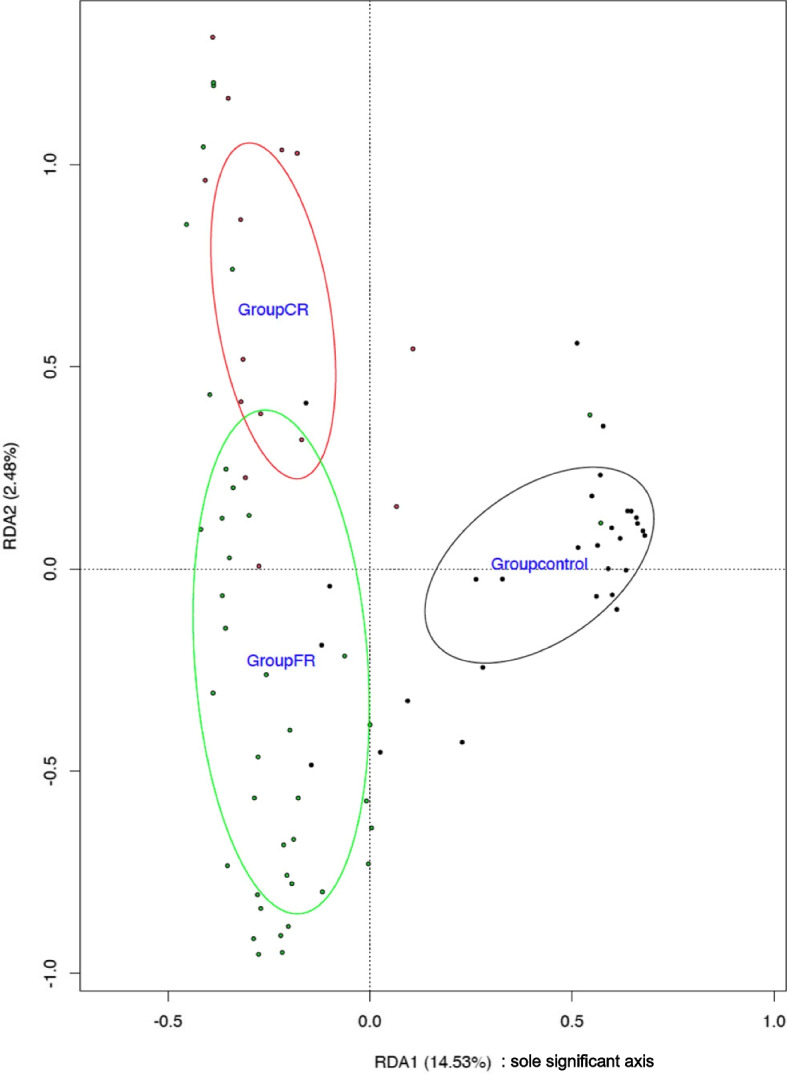
Redundancy analysis at genus level for microbiota composition in dogs based on disease status. FR: fungal rhinitis, CR: chronic idiopathic rhinitis

Redundancy analysis (RDA) showed that only disease status (*p* = 0.002; Adjusted R [2] 0.142) contributed significantly to the variability of the microbiota (explaining 14.5% of the variance, Fig. 2).

#### Intrinsic diversity values and β-diversity

**Fig. 3:**
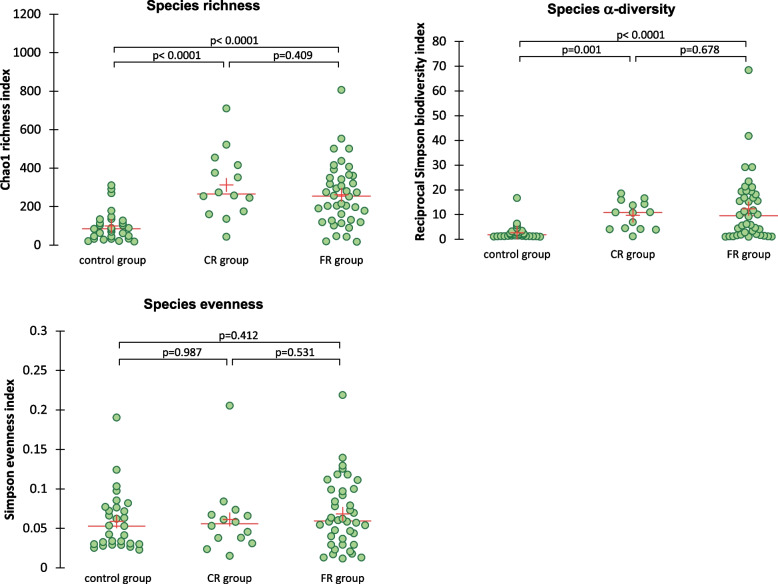
Intrinsic diversity values comparing healthy dogs and dogs with chronic nasal diseases. FR: fungal rhinitis, CR: chronic idiopathic rhinitis

**Fig. 4:**
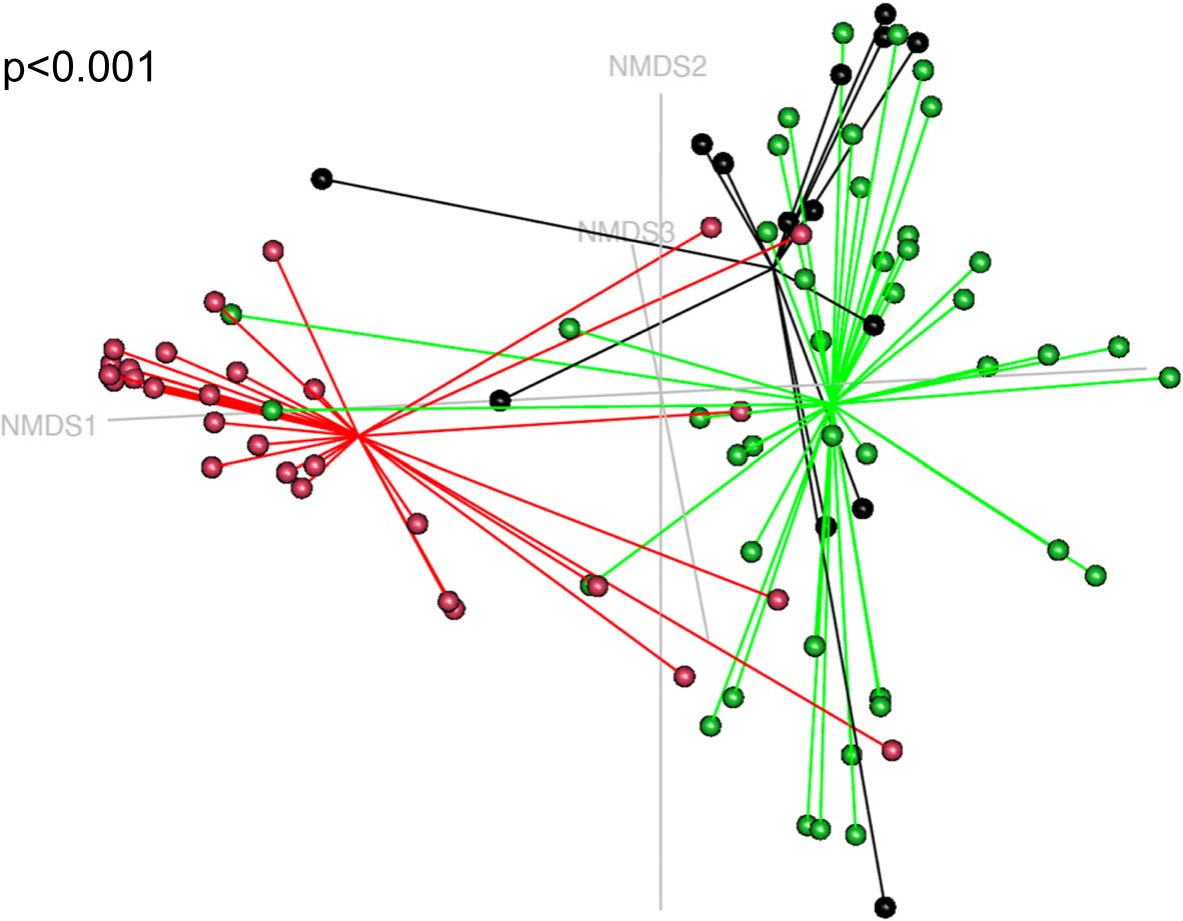
Non-metric multidimensional scaling (NMDS) ordination of nasal microbiota communities using Bray–Curtis. Comparison between fungal rhinitis (FR) group (green), chronic idiopathic rhinitis (CR) group (black) and control group (red). CR group versus FR group *p* = 0.054, CR group versus control group *p* < 0.001, FR group versus control group *p* < 0.001. Stress value = 0.09800267

Good’s coverage, species richness and α-diversity were significantly different between healthy dogs and dogs with chronic nasal diseases (Fig. 3). There was no difference in evenness. The non-metric multidimensional scaling graph of the β-diversity shows a clustering for the group of healthy dogs separating them from the diseased dogs (Fig. 4).

#### Differences in relative abundances: FR group versus control group

**Fig. 5:**
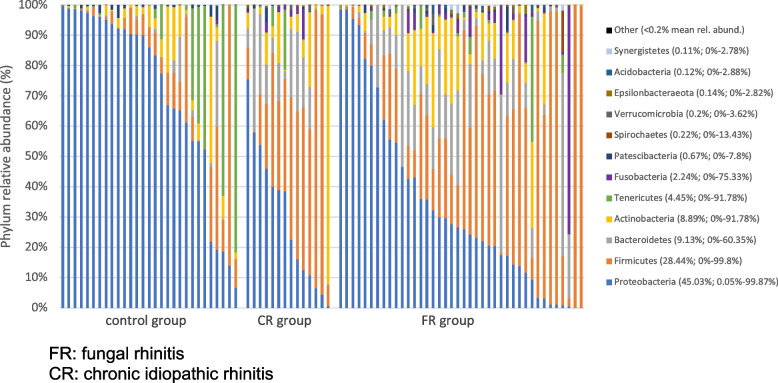
Composition of nasal microbiota at phylum level in the study population. FR: fungal rhinitis. CR: chronic idiopathic rhinitis

**Fig. 6:**
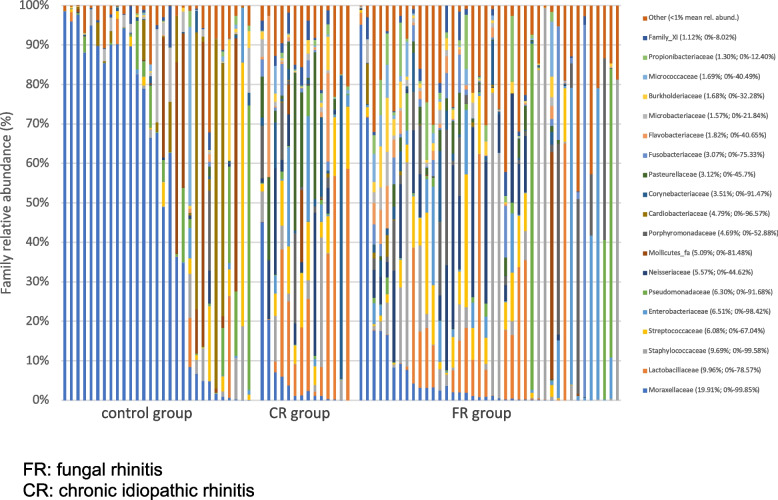
Composition of nasal microbiota at family level in the study population. FR: fungal rhinitis. CR: chronic idiopathic rhinitis

Mean relative abundances at phylum and family level are represented in Figs. 5 and 6. Table 2 shows the mean relative abundances of most abundant OTU, annotated to the levels of phylum, family and genus. The relative abundance in the Proteobacteria phylum was significantly lower in the FR group, compared with control dogs. This lower abundance in Proteobacteria was associated with a major and significant lower abundance in *Moraxella* (family Moraxellaceae) and *Suttonella* (family Cardiobacteriaceae) together with an increase of *Conchiformibius* (family Neisseriaceae). Other significant differences in the FR group compared with healthy dogs included an increase in the Firmicutes phylum with associated family Lachnospiraceae, an increase in the Bacteroidetes phylum with associated family *Porphyromonas*, an increase in Actinomycetaceae (phylum Actinobacteria), and finally a decrease in an unclassified genus of the Mollicutes class (phylum Tenericutes).

#### Differences in relative abundances: CR group versus control group

Similar alterations were also observed between the CR and control groups especially the decrease in Proteobacteria due to the decrease in *Moraxella* in contrast to an increase in the phyla Firmicutes and Bacteroidetes. Specific changes were also noted, such as an increase in the genera *Lactobacillus* (family Lactobaciliaceae) and *Lactococcus* (family Streptococcaceae) among the Firmicutes phylum. An increase in the genus *Neisseria* (family Neisseriaceae, phylum Proteobacteria) and the family Flavobacteriaceae (phylum Bacteroidetes) was also noted.

##### Differences in relative abundances: FR group versus CR group

Between the FR and CR groups specifically, three significant differences were present: a higher relative abundance of *Pasteurella* and unclassified genus of the Pasteurellaceae family (both family Pasteurellaceae) as well as *Gemella* (family Bacillales_Family_XI) in the CR group compared to the FR group.

#### Linear discriminant analysis effect size scores

**Fig. 7:**
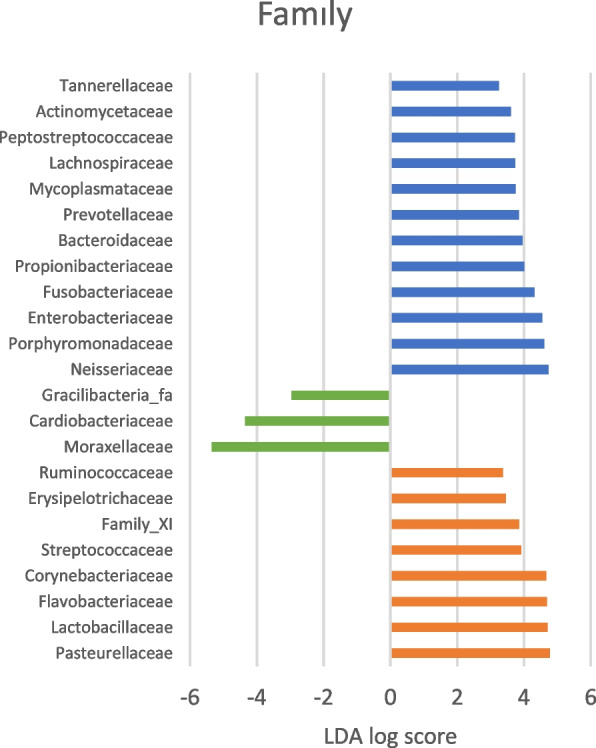
Linear discriminant analysis (LDA) effect size (LEfSe) of Illumina sequencing datasets based on 16S rRNA gene sequences. Differentially abundant OTUs were detected (*p* = 0.05, LDA score > 3.0) at family level. FR group (blue), control dogs (green) and CR group (orange)

In Fig. 7, LEfSe scores indicate bacterial taxa that were mainly present in the different groups of the study population and shows that the highest number of specific taxa are found in the FR group, followed by the CR and the control group.

### Dogs with fungal rhinitis

#### Comparison between diagnosis and cure

**Fig. 8:**
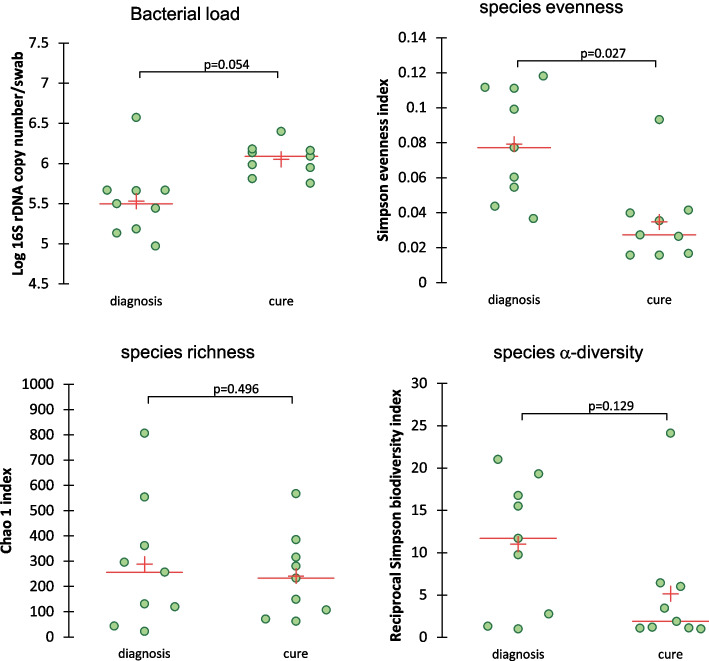
Bacterial load and intrinsic diversity values in dogs with fungal rhinitis at diagnosis and cure

The microbial composition of the 9 dogs at diagnosis and cure at family level are represented in Fig. 1. Only 1 dog (dog 2) recovered a high relative abundance of Moraxellaceae at cure. In 2 dogs (dogs 8 and 9) the NM was very similar to the one observed at diagnosis and in 5 dogs the microbiota was dominated (> 50%) by a single family: Porphyromonadaceae (dog 1), Spirochaetaceae (dog 3), Staphylococcaceae (dogs 4 and 6) and Enterobacteriaceae (dog 5). In the remaining dog (dog 7), a more heterogeneous composition was observed which was very different from its composition at diagnosis. No difference in bacterial load was observed between the two timepoints (Fig. 8). Among the intrinsic diversity values, only species evenness differed and was found to be lower at cure compared with the time of diagnosis (Fig. 8). No significant differences in relative abundances were found. NMDS plot did not show a specific pattern. Based on analysis of molecular variance (AMOVA; *p* = 0.202) and analysis of molecular variance homogeneity (HOMOVA; *p* = 0.905) beta-diversity and beta-dispersion were not different either.

#### Effect of treatment on the NM

Among dogs within the FR group, 11 were treated with systemic antimicrobials at the time of sampling while 29 dogs had not been receiving antimicrobials within at least the 2 previous weeks. Treated dogs were receiving amoxycillin clavulanic acid (*n* = 7), marbofloxacin (*n* = 1), marbofloxacin associated with azithromycin (*n* = 1), doxycycline (*n* = 1) or metronidazole (*n* = 1). Ten dogs were receiving an oral antifungal treatment (itraconazole, *n* = 8; ketoconazole, *n* = 1; or fluconazole, *n* = 1) at the time of sampling.

For these two types of treatments, there was no significant effect on the variance (redundancy analysis), there were no differences at the level of the intrinsic diversity values, β-diversity or relative abundances at family, genus or species level. There was also no difference in bacterial load between the dogs receiving and not receiving antimicrobial or antifungal treatment.

## Discussion

The present study is the first to describe the NM in dogs with fungal rhinitis using next generation sequencing methods. Our data showed that both fungal rhinitis and chronic idiopathic rhinitis were associated with common major alterations of the NM. These alterations were characterized by a significant lower abundance in Proteobacteria, mainly due to a lower abundance in *Moraxella* while more minor differences were specific either to fungal rhinitis or chronic idiopathic rhinitis. In most dogs with cured fungal rhinitis, the NM was still different from what we consider a healthy profile. Neither antimicrobial nor antifungal treatment appears to have a significant effect on the NM in dogs with fungal rhinitis.

We showed that the NM in the healthy group was mostly dominated by the phylum Proteobacteria. This is in agreement with results of previous publications showing that the phyla Proteobacteria represents around 50 to 80% of the total bacterial population independently from age, breed or environment [8–10, 37]. Other common phyla detected in healthy dogs in this study included Firmicutes, Tenericutes, Actinobacteria, and Bacteroidetes. This is also similar to previous publications although their frequency order may vary according to the study [8–10, 37].

Like in previous studies, the Proteobacteria population was dominated by the family Moraxellaceae, and the genus *Moraxella*, followed by several other bacterial families at considerably lower levels [8–10, 37]. However, not all healthy dogs had a microbial profile dominated by Moraxellaceae. Profiles dominated by Cardiobacteriaceae (phylum Proteobacteria), although in a much smaller amount, were also present. Cardiobacteriaceae was also one of the most frequently identified families in healthy dogs in previous studies [10]. Finally, some healthy dogs presented a more heterogenous profile, which was also the case in previous studies [10]. It can be hypothesized that, as it has been described in humans [1, 38], different healthy profiles exist, some of them being dominated by a particular bacterial taxon (e.g. Moraxellaceae, Cardiobacteriaceae) and others being more heterogeneous. The inter-individual variability of the nasal microbiota in healthy dogs has been described previously [9] and reflects the concept of a personalized microbiota [39, 40]. Moreover, the microbiota constantly undergoes changes of resident and transient micro-organisms in response to internal and external factors [4, 12]. These factors may include the host and its local immune system, the inhaled particle-laden air, as well as atmospheric physical and chemical parameters. This is particularly true for the NM in dogs that interacts closely with the external environment which complicates the study of the NM in dogs. Based on the results of the present study and a previous study of the same group [12], facial conformation (particularly brachycephalic breeds) and disease status are two factors able to significantly influence the NM in dogs.

Both fungal rhinitis and chronic idiopathic rhinitis were associated with common major alterations of the resident nasal microbiota compared with healthy dogs. The most noticeable modification in both diseases was the marked lower relative abundance of the phylum Proteobacteria (± 50% reduction) and the associated family Moraxellaceae and genus *Moraxella* (± 90% reduction). Such a low relative abundance of *Moraxella* in dogs with chronic nasal diseases had already been described in the study by Tress and others [10] who compared the NM in healthy dogs to dogs with nasal neoplasia and chronic rhinitis. In children, nasopharyngeal *Moraxella*-dominated profiles have been described to be more stable and associated with a lower frequency of upper respiratory tract infections [38]. Altogether, these findings might propose *Moraxella* as a guarantor of nasal health. In dogs with chronic nasal disease, establishment of opportunistic species or overgrowth of some strains of the resident flora could overwhelm *Moraxella*, leading to a dysbiotic profile. It should be pointed out that a lower amount of *Moraxella* has also been observed in healthy brachycephalic dogs compared to other breed types [12], although to a much lesser degree compared to the current data in dogs with nasal disease. This suggests that the relative abondance of *Moraxella* is at least partly dependent on facial conformation and/or air distribution strategy, rather than being exclusively associated with disease.

In dogs with nasal diseases, the inter-individual variability of the NM was obvious. This has also been described in humans with chronic rhinosinusitis and tend to complicate interpretation of results in microbiome studies [41]. Due to disruption of the normal protective mechanisms, it is expected that secondary bacterial infection can occur during nasal disease. The reasons why certain specific bacteria get the upper hand and develop is unknown. It is possible that local intranasal physical and chemical characteristics, personal nasal microbiota before the development of disease, host genetics, local host immunity and the type of nasal disease play a role.

The most noticeable alterations specific to dogs with fungal rhinitis included the higher abundance of genera such as *Staphylococcus, Conchiformibius*, *Escherichia_Shigella*, *Porphyromonas* and *Fusobacteria*, some taxa frequently reaching abundances of > 50% in individuals with fungal rhinitis. In healthy dogs, the same genera were also present but in small abundances, suggesting that fungal infection allows their particular development. In dogs with CR, the most noticeable alterations were different, in particular with a higher abundance of *Pasteurella* and *Lactobacillus*, underlining the fact that FR and CR are two distinct diseases each causing unique alterations of the NM. Whether these types of dysbiosis are the consequences of the alterations associated with FR and CR or if they play an active role in the development of the disease remains to be determined.

In human beings, bacterial co-infections have been suggested to influence the development and persistence of clinical symptoms in patients with paranasal sinus *A. fumigatus* fungal balls [42, 43]. In a sheep model of sinusitis [26], inoculation of *A. fumigatus* resulted in the formation of a fungal biofilm only when co-inoculated with certain bacterial strains (*S. aureus, S. epidermidis, P. aeruginosa*). In the current study, in dogs with FR at diagnosis, genera such as *Staphylococcus* (*n* = 5), *Pseudomonas* (*n* = 2), *Porphyromonas* (*n* = 3), *Escherichia_Shigella* (*n* = 3), *Conchiformibius* (*n* = 4) and *Lactobacillus* (*n* = 3) represented the major part of the bacterial population (> 50%) in half (21/40) of the dogs, and might play an active role in the establishment, persistence and recurrence of fungal infection, either by causing epithelial inflammation and injury, or by metabolite cross-talk, and/or by modifying the immune response of the host to the fungus.

A longer follow-up in dogs with FR would allow to verify the association between these specific taxa and either resolution or recurrence of the fungal infection.

In dogs with fungal rhinitis at the time of cure, the NM was globally highly unpredictable. Amongst these 9 dogs, only one dog recovered a microbial composition with a high prevalence of Moraxellaceae. This dog needed 3 infusion protocols to reach cure, meaning he was the dog with the longest timeframe (3 months) between the collection of the two swabs. This may suggest that the NM needs more time to return to his “healthy state”. Another possibility would be that in some individuals the NM returns to his pre-infection state while in others it continues to shift toward a new and different bacterial community, a scenario that has already been described in humans with chronic rhinosinusitis after sinus surgery [44].

The Pasteurellaceae and Lactobacillaceae were much more prominent in dogs with chronic idiopathic rhinitis in this study. An increase in Pasteurellaceae was earlier reported in dogs with chronic rhinitis and nasal neoplasia [10]. In the current study, 2 dogs were colonized with a high amount of *Pasteurella multocida*, which was absent in the nose of healthy dogs in the current study. This species is considered a primary pathogen in swine [45] but is also described as an opportunistic pathogen in human and veterinary medicine. The role of *P. multocida* as a primary or opportunistic pathogen in dogs with chronic idiopathic rhinitis is currently unknown but deserves to be considered.

Lactobacilli are commensals of the gastrointestinal and female genital tract [46, 47] also used as probiotic strains [48, 49] or feed additives [50, 51] in dogs. However, it seems unlikely that Lactobacillus play a role in the pathogenesis of CR in dogs since they are uncommonly depicted as an opportunistic pathogen [52], and were not reported to be elevated in dogs with CR in the study by Tress and others [10] or in humans with chronic rhinosinusitis.

Results of the present study showed that systemic antimicrobials do not seem to significantly influence the NM in dogs with fungal rhinitis. In human beings with chronic rhinosinusitis, contradicting results have been published with variable effect on the diversity, evenness and bacterial burden [41, 44, 53–55]. Another study in dogs with nasal neoplasia also showed that pretreatment with antibiotics did not significantly alter the NM [10]. The lack of effect could be due to a small concentration of drug reaching the nasal mucosa or a high resilience [44] of the NM to short-term antibiotic treatments and makes the use systemic antimicrobial questionable in canine chronic nasal diseases.

The influence of age and bodyweight on the NM is unclear in dogs, based on previous studies this influence seems weak or absent [9, 10, 12]. Facial conformation however has been associated with significant changes of the NM in healthy dogs [12]. These changes were mostly present in dogs of brachycephalic breeds compared to other breed types, but minor variations were also observed between dolichocephalic and terrier breeds. Antibiotic pretreatment has also been reported to influence NM at varying degrees in humans and dogs [10, 41, 53–55] and the possible influence of antifungal treatment is unknown. For these reasons we decided to take age, bodyweight, breed type (meso-/dolichocephalic or terrier breed) and treatment status (antibiotic and antifungal) into account along with disease status (FR, CR or control group) for the RDA, as we believe these individual factors were the most likely to influence the variance in microbiota community composition. Sex and living environment (rural versus industrial regions) were considered unlikely to influence the NM [9, 10, 12].

The present study is essentially descriptive. We did not measure local microenvironmental parameters such as intranasal pH, humidity or temperature. Neither did we determine viral or fungal populations and the host immune response, preventing interpretation of the NM in light of these parameters.

Another limitation concerns the size of the group, essentially the dogs with chronic idiopathic rhinitis, which is moreover a heterogeneous disease, of unclear and possibly variable etiology.

A long-term follow-up was not performed in dogs with fungal rhinitis to evaluate the evolution of the NM in the presence or absence of relapse or recurrence of the disease.

And finally, the present study was not designed to assess the effect of antimicrobial treatment: the molecules and duration of treatment were not standardized and the number of dogs in the treated group was small. This could possibly explain why we failed to show statistical differences between treated and non-treated groups.

In conclusion, in dogs with chronic nasal diseases such as FR and CR, major alterations are present compared to healthy dogs while more subtle but significant differences might distinguish both diseases. Most dogs with fungal rhinitis probably did not recover their core microbiota at cure. The NM in dogs with fungal rhinitis at cure was unpredictable and a longer follow-up is needed to draw a conclusion. The present study lays the first groundwork to the realization and comprehension of the complex interactions between the nasal microbiota and nasal *Aspergillus fumigatus* infection in dogs. Further studies are warranted to discover if modulation of the nasal microbiota might be an interesting perspective for the treatment of this disease.

## Methods

### Study sample

Client-owned dogs with a diagnosis of fungal rhinitis (FR group) or chronic idiopathic rhinitis (CR group) were prospectively recruited. The CR group was mainly included in order to differentiate changes in NM due to the presence of inflammation from changes in NM specifically associated with fungal rhinitis.

A control group of healthy dolichocephalic dogs, age and breed matched with the FR group, was also recruited. Part of the dogs with fungal rhinitis that were treated and cured were examined at checkup (cured FR group).

Diagnosis of fungal rhinitis was based on the presence of compatible clinical signs and per-endoscopic identification of fungal plaques with turbinate destruction. Additional diagnostic procedures consisted of computed tomography of the head, histopathology and fungal culture or polymerase chain reaction (PCR). All dogs were treated with endoscopic debridement of the fungal plaques followed by a 15-min enilconazole infusion protocol [56]. Control rhinoscopy was performed 3 to 6 weeks after treatment. Cure was based on resolution of clinical signs and absence of fungal plaques. As a non-negligible amount of the dogs included in the FR group were treated with antimicrobial and/or antifungal treatment at the time of sampling, the potential effect of these treatments on the NM was also investigated.

Diagnosis of chronic idiopathic rhinitis was based on compatible clinical signs, endoscopic and/or histopathologic lesions. Other nasal diseases such as fungal rhinitis, neoplasia, oronasal defect or foreign body were excluded based on different examinations including endoscopy, computed tomography of the head, histopathology, culture or PCR results from nasal samples.

All healthy dogs were exempt of clinical signs and had a normal clinical examination and blood work.

In all dogs, questions were asked concerning ongoing local or systemic medical treatment. Except for dogs in the control group, the presence of antimicrobial or anti-inflammatory treatment before or at the time of sampling was not an exclusion criterion.

### Sample collection

This study was approved by the animal ethical committee (N° 16–1854, 27/10/2016) and all samples were obtained with the consent of the owners.

For sample collection, dogs were premedicated with a combination of butorphanol (Butomidor®, Richter Pharma) and medetomidine (Medetor®, CP-Pharma) intravenously. Propofol (Propovet®, Zoetis) on demand was used for induction. Under general anesthesia, to prevent sample contamination, a sterile speculum was inserted into the nare to allow the passage of a sterile swab (Copan®, FLOQSwabs™, 553C, Brescia, Italy) in the distal third of the nasal cavity. Sample collection was performed either by EV, FB or CC. In case of unilateral fungal rhinitis, the affected nasal cavity was sampled. In diseased dogs, sample collection was performed before rhinoscopy. The nasal mucosa was brushed using three careful circular movements before withdrawal of the swab through the speculum. The tip of the saw was cut and stored in a sterile cryotube and banked at -80 °C until further analyses.

### DNA extraction and high throughput sequencing

Based on the manufacturer’s instructions, total bacterial DNA was extracted from the nasal swabs with the DNEasy Blood and Tissue kit (QIAGEN Benelux BV; Antwerp, Belgium). Spectrophotometry (NanoDrop ND-1000, Isogen, De Meern, The Netherlands) was used for total DNA concentration measurement and purity evaluation.

After DNA extraction from samples, quantification of the bacterial load was performed with a quantitative real-time PCR targeting the V2-V3 region of the 16S rRNA gene with the following primers: forward (5’-ACTCCTACGGGAGGCAGCAG-3’) and reverse (5’-ATTACCGCGGCTGCTGG-3’) as previously described [57]. The standard curve was based upon tenfold dilution of a quantified PCR product. This PCR product was purified (Wizard® SV Gel and PCR Clean-Up System, Promega, Leiden, The Netherlands), quantified with PicoGreen targeting double-stranded DNA (Promega).

For bacterial identification, bacterial 16S rRNA gene amplicons were generated via amplification of the V1-V3 hypervariable regions of the 16S rRNA gene using the following primers: forward (5’-GAGAGTTTGATYMTGGCTCAG-3’) and reverse (5’-ACCGCGGCTGCTGGCAC-3’) and Illumina overhand adapters. The DNA was purified with the Agencourt AMPure XP beads kit (Beckman Coulter; Pasadena, CA, USA) and submitted to a second PCR round for indexing, using the Nextera XT index primers 1 and 2. A final quantification, performed by quantitative PCR, of each sample in the library was performed using the KAPA SYBR" FAST qPCR Kit (KapaBiosystems; Wilmington, MA, USA) before normalization, pooling and sequencing on a MiSeq sequencer using V3 reagents (Illumina; San Diego, CA, USA). Positive control using DNA from 20 defined bacterial species and a negative control (from the PCR step) were included in the sequencing run.

### Amplicon profiling analysis

Alignment and clustering were done with MOTHUR software package (v1.41.0) with an OTU clustering distance of 0.03 and based on the SILVA database (V1.32) of full-length 16S rRNA gene sequences. Vsearch algorithm was used for chimera detection [58]. After the chimera removal, reads corresponding to chloroplastic and mitochondrial 16S rRNA genes and reads whose taxonomic assignation fall outside the bacterial kingdom are removed during the cleaning process. From 16,220,278 raw reads, we obtained 14,714,808 reads after cleaning (length and sequence quality). Finally, we retained 6000 reads (median 5999 reads per sample) to adjust for uneven sequencing depth across samples. All biosample raw reads were deposited at the National Center for Biotechnology Information (NCBI) and are available under de Bioproject ID PRJNA841569.

#### Alpha- and beta diversity

Subsample data sets including bacterial richness, evenness and α-diversity were obtained with MOTHUR at species level using the Chao1 index, Simpson index-based measure and the inverse Simpson’s index respectively. Beta-diversity at species level was assessed with MOTHUR using a dissimilarity matrix of Bray–Curtis. Non-metric multidimensional scaling plots for visual assessment were performed based on a Bray–Curtis dissimilarity matrix at species level with Rstudio (R v1.2.5033 package vegan v2.5–6 and ggplot2 v3.3.0) to represent the β-diversity between groups (FR versus CR versus control group, diagnosis versus cure within the FR group, treated versus non-treated dogs within the FR group).

### Statistical analysis

#### Redundancy analysis

A RDA on values at genus level was performed to evaluate the relationships between the NM and the different potential explanatory variables (age, bodyweight, breed type, disease status and, among the FR group, antimicrobial or antifungal treatment status) that could influence/shape it. Forward selection was conducted to select significant variables using the “ordiR2step” function (with adjusted R2 coefficient) from the vegan package [59].

#### Relative abundances of taxa

A Kruskal–Wallis test with Benjamini-Hotchberg FDR correction followed by Tukey’s multiple comparison test in STAMP (v2.1.3) were used to identify differences in relative abundance at phylum, family, genus and species level (FR versus CR versus control group and treated versus non-treated dogs within the FR group).

#### Alpha- and beta diversity

Bacterial richness, evenness, α-diversity, good’s.coverage index and bacterial load were compared between the three groups (FR versus CR versus control group and treated versus non-treated dogs within the FR group) using a Kruskal–Wallis test and Dunn post-hoc test with Bonferroni correction or a Wilcoxon rank test for paired samples (diagnosis versus cure within the FR group). These analyses were performed using XLstat (2020.5.1, Addinsoft, Paris, France). Differences were considered significant for a p-value < 0.05.

Beta-diversity was estimated with AMOVA (analysis of molecular variance; 10,000 iterations) and beta-dispersion was assessed with HOMOVA (analysis of molecular variance homogeneity; 10,000 iterations).

#### Linear discriminant analysis (LDA) effect size (LEfSe) score

LEfSe was performed to detect differences in bacterial composition between groups (FR versus CR versus control group) at phylum, family, genus and species level with MOTHUR (significant for an LDA score > 3.0 [60].

## Data Availability

All sample raw reads associated with this study have been deposited at the National Center for Biotechnology Information (NCBI) under the accession number PRJNA841569.

## Abbreviations

NM: Nasal microbiota
FR: Fungal rhinosinusitis
CR: Chronic idiopathic rhinitis
OTU: Operational taxonomic unit
rRNA: Ribosomal ribonucleic acid
DNA: Deoxyribonucleic acid
rDNA: Ribosomal deoxyribonucleic acid
PCR: Polymerase chain reaction
qPCR: Quantitative polymerase chain reaction
RDA: Redundancy analysis
LEFSe: Linear discriminant analysis effect size
NMDS: Non-metric multidimensional scaling
AMOVA: Analysis of molecular variance
HOMOVA: Homogeneity of molecular variance
LDA: Linear discriminant analysis
